# Effects of Immediate Aversive Stimulation on Haloperidol-Induced Catalepsy in Rats

**DOI:** 10.3389/fnbeh.2022.867180

**Published:** 2022-04-11

**Authors:** Isabelle Waku, Adriano E. Reimer, Amanda R. de Oliveira

**Affiliations:** ^1^Department of Psychology, Federal University of São Carlos (UFSCar), São Carlos, Brazil; ^2^Institute of Neuroscience and Behavior (INeC), Ribeirão Preto, Brazil

**Keywords:** paradoxical kinesia, emotional state, dopamine, D2 antagonist, anxiety, animal model, Parkinson’s disease

## Abstract

In animal models, the administration of the dopaminergic D2 antagonist haloperidol affects the nigrostriatal pathway, inducing catalepsy, a state of immobility similar to Parkinson’s disease (PD) bradykinesia and akinesia. In PD, the motor impairments are due to difficulties in selecting and executing motor actions, associated with dopamine loss in basal ganglia and cortical targets. Motor and affective limbic networks seem to be integrated *via* a striato-nigro-striatal network, therefore, it is not surprising that the motor impairments in PD can be influenced by the patient’s emotional state. Indeed, when exposed to aversive stimuli or life-threatening events, immobile patients are capable of performing sudden movements, a phenomenon known as paradoxical kinesia. Thus, the present study investigated the effects of unconditioned and conditioned aversive stimulation on haloperidol-induced catalepsy in rats. First, male Wistar rats received intraperitoneal administration of saline or haloperidol (1 or 2 mg/kg) and were evaluated in the catalepsy bar test to assess the cataleptic state induced by the different doses of haloperidol over time. Next, we evaluated the effects of two types of unconditioned aversive stimuli–100 lux light (1 and 20 s) or 0.6 mA footshock (1 s)–on the catalepsy. Finally, we evaluated the effects of light conditioned stimuli (Light-CS), previously paired with footshocks, on the cataleptic state. Catalepsy was observed following haloperidol 1 and 2 mg/kg administration. Exposure to footshocks, but not to light, significantly reduced step-down latency during the catalepsy test. Although unconditioned light did not affect catalepsy, paired Light-CS did reduce step-down latency. Here, we have provided evidence of face validity for the study of paradoxical kinesia. In addition to demonstrating that immediate exposure to an aversive stimulus is capable of disrupting the cataleptic state, our findings show that haloperidol-induced catalepsy seems to be differently influenced depending on the modality of aversive stimulation. Our data suggest that the selective recruitment of threat response systems may bypass the dysfunctional motor circuit leading to the activation of alternative routes to drive movement.

## Introduction

Haloperidol is an antipsychotic drug that acts as a D2 receptor antagonist and can induce unwanted extrapyramidal side effects, such as akinesia and rigidity of movement (Reynolds, 1992; Muticã et al., 2016). In rodents, haloperidol can induce catalepsy, a state of immobility similar to Parkinson’s disease (PD) bradykinesia and akinesia (De Ryck et al., 1980; Lorenc-Koci et al., 1996; Wadenberg et al., 2001; Kulkarni et al., 2009). The degree of catalepsy is commonly evaluated by the catalepsy bar test, which quantifies the time it takes for the animal to correct an externally imposed posture (Sanberg et al., 1988; Melo et al., 2010; Colombo et al., 2013; Barroca et al., 2019). Given the simplicity and ease of use of the test, haloperidol-induced catalepsy often serves as a useful animal model for the study of parkinsonism and screening of potential antiparkinsonian compounds (Waku et al., 2021).

Parkinson’s disease motor symptoms such as tremors, rigidity, postural instability, and bradykinesia can be comorbid with non-motor manifestations such as cognitive and emotional impairments (Walsh and Bennett, 2001; Parkinson, 2002; Jankovic, 2008; Chagas et al., 2009; Dickson, 2012; Broen et al., 2016). Although a link between increased anxiety and worsening of motor symptoms has been suggested (Dissanayaka et al., 2010), a relationship between increased anxiety and decreased motor symptoms has also been observed (Bonanni et al., 2010; McDonald et al., 2015). Despite presenting severe motor symptoms, some patients may occasionally perform normal, fast, and precise movements or tasks. This phenomenon is known as paradoxical kinesia and it seems to be triggered by intense emotions such as fear or anger or even by sudden visual stimuli (Glickstein and Stein, 1991; Schlesinger et al., 2007; Jankovic, 2008; Bonanni et al., 2010). Paradoxical kinesia is of particular interest, as it suggests that alternative motor pathways could be partially or fully functional and that clinical interventions targeting those circuits could reestablish or improve mobility in PD patients (Naugle et al., 2010; Day, 2014; Potocanac and Duysens, 2017; Duysens and Forner-Cordero, 2018).

Dopamine deficiency in the nigrostriatal pathway is the major neurochemical abnormality in PD (Lotharius and Brundin, 2002; Moore, 2003; Martinez et al., 2013; Blaszczyk, 2017). PD progression can also lead to important neuronal loss in the ventral tegmental area, the starting point of the mesocorticolimbic dopaminergic pathway and a critical substrate involved in emotion and cognition modulation (Walsh and Bennett, 2001; Remy et al., 2005; Alberico et al., 2015). Since the mesocorticolimbic pathway modulates fear/anxiety-related responses (De Oliveira et al., 2009, 2011, 2017; De Souza Caetano et al., 2013), it is not surprising that the patient’s emotional state can influence PD’s motor impairments. The role of dopaminergic mechanisms in regulating adaptive responses to threatening situations is widely studied and appears to depend on the type of aversive or stressful stimuli triggering the coping reaction (De Oliveira et al., 2014; Muthuraju et al., 2014; Brandão et al., 2015). The dopaminergic modulation of adaptive responses to stressful situations is, however, less studied in the context of catalepsy. Therefore, it is of great interest to study the interplay between catalepsy and emotional states (Melo et al., 2010; Colombo et al., 2013; Melo-Thomas and Thomas, 2015; Barroca et al., 2019; Ihme et al., 2020).

We have demonstrated that haloperidol reduces alarm calls emission during re-exposure to an aversive context but not to footshocks, nor during the catalepsy or open field tests, implicating heterogeneous participation of dopaminergic mechanisms contingent on the nature of the aversive stimulus (Colombo et al., 2013). Next, we verified that exposure to aversive stimuli such as open field, elevated plus-maze, and footshocks does not affect, while context-aversive conditioning seems to potentiate catalepsy (Barroca et al., 2019). Once again, the results suggest that different aversive situations, unconditioned or conditioned, seem to influence differently the cataleptic state caused by haloperidol (Barroca et al., 2019). More broadly, it can be presupposed that the intensity of motor symptoms observed in PD patients may vary according to the type of aversive situation the individual is faced with.

Previous studies, however, have not assessed how aversive stimulation directly impacts catalepsy, especially in the context of the paradoxical kinesia phenomenon. Seeking a model for the study of paradoxical kinesia while continuing to investigate the influence of fear/anxiety states on catalepsy, we presented the aversive stimuli at the exact moment of the catalepsy test. We hypothesized that haloperidol-induced catalepsy in rats could be disrupted or at least attenuated with the immediate exposure of the animals to aversive stimulation. Results in this direction could contribute to an animal model for the study of paradoxical kinesia, favoring advances in the understanding of the neural bases associated with the interactions between aversive states and parkinsonism and contributing to better therapeutic strategies. In fact, results of studies that investigated paradoxical kinesia using animal models pointed to a reduction of haloperidol-induced catalepsy when an auditory cue (Clark et al., 2009), deep brain stimulation of the inferior colliculus (Melo-Thomas and Thomas, 2015), or appetitive 50-kHz ultrasonic vocalizations (Tonelli et al., 2017, 2018) were presented during the catalepsy test.

Here, in order to investigate the influence of conditioned and unconditioned aversive stimulation on haloperidol-induced catalepsy, distinct groups of rats were exposed to light and footshock stimuli. Exposure to inescapable footshocks activates nociceptors interfering with the pain motivational system (Bolles and Fanselow, 1980). On the other hand, in studies on the neurobiology of fear/anxiety, light is generally used as a conditioned aversive stimulus (CS) (De Oliveira et al., 2006, 2017; Cassaday and Thur, 2015; Pezze et al., 2016). However, depending on the duration and/or intensity, light can be aversive by itself, with the escape from illuminated areas being considered an innate response of rodents (Crawley and Goodwin, 1980; Bourin and Hascoet, 2003; Reis et al., 2004; Saito and Brandão, 2016). So, initially, we assessed catalepsy induced by different doses of haloperidol over time; then we investigated the effects of two types of unconditioned aversive stimuli–100 lux light or 0.6 mA footshock–on haloperidol-induced catalepsy; and lastly, we investigated the effects of light-CS on haloperidol-induced catalepsy. Our results show that, when subjected to appropriate aversive stimulation, i.e., footshocks or light-CS, haloperidol-induced catalepsy in rats can be disrupted, suggesting emotional modulation of the motor system and providing a promising animal model to study paradoxical kinesia.

## Materials and Methods

### Subjects

One hundred and two naïve male Wistar rats (300–350 g; 8–12 weeks) were supplied by the animal facility of the Federal University of São Carlos (UFSCar). Rats were housed in groups of four per cage (polypropylene boxes, 40 cm × 33 cm × 25 cm), under a 12/12 h light/dark cycle (lights on at 07:00 h), at 23–25°C, with constant access to food and water. Before the experiments, the animals were allowed to habituate to the laboratory conditions for at least 72 h. Experiments were conducted during the light phase of the cycle. All procedures were carried out in accordance with the National Council for Animal Experimentation Control and were approved by the Committees for Animal Care and Use of the Federal University of São Carlos (protocol no. 5413130319).

### Drugs

The D2 antagonist haloperidol (Tocris Bioscience, Bristol, United Kingdom) was used to induce cataleptic states. Haloperidol was dissolved in physiological saline (0.9%) containing 2% Tween 80 to obtain concentrations of 1 and 2 mg/ml. Injections were administered intraperitoneally in a constant volume of 1 ml/kg, 15 min before the beginning of the tests. The control group received an equivalent volume of physiological saline containing 2% Tween 80 (vehicle). The drug doses were based on previous studies (Colombo et al., 2013; Barroca et al., 2019; Waku et al., 2021).

### Haloperidol-Induced Catalepsy Test

Rats were placed in a cage (26 cm × 20 cm × 20 cm) with a white painted metal ceiling and back and sidewalls, a transparent glass door, and a grid floor with thirteen 5 mm diameter metal rods (1.5 cm apart). One wall had a white lamp attached. Footshocks were delivered through the cage floor *via* a constant current generator. The cage was enclosed in a sound-attenuating aluminum chamber (65 cm × 43 cm × 45 cm). For the catalepsy test, the cage contained a glass bar (5 mm in diameter) positioned horizontally 8 cm above the floor. The catalepsy test consisted of carefully positioning the rat in an upright position, with forepaws on the horizontal glass bar, while their hind paws remained on the floor. The duration of catalepsy was measured as the latency to step-down from the horizontal bar. For Experiment 1, rats were tested for catalepsy 15, 30, 45, 60, 75, 90, 105, and 120 min after vehicle or haloperidol administration. For Experiments 2 and 3, rats were tested for catalepsy 75 and 90 min after haloperidol administration. The maximum time allowed for the animals to remain on the bar was fixed at 5 min for Experiment 1, and 3 min for Experiments 2 and 3. During the interval between catalepsy tests, animals remained in the experimental chamber.

### Experiment 1: Evaluation of Haloperidol-Induced Catalepsy Over Time in Rats

For Experiment 1, a total of 36 animals were sampled. Rats were subjected to catalepsy tests receiving no aversive stimulation, to assess the efficacy of haloperidol in inducing catalepsy over time. At the beginning of the experiment, the animals received an intraperitoneal injection of vehicle or haloperidol 1 or 2 mg/kg (*n* = 12 per group). Next, animals were transferred to the experimental chamber and 15 min later, the first catalepsy test was performed and the step-down latency was measured. Each animal underwent a total of eight trials (15 min inter-trial interval). The experiment lasted a total of 120 min. Based on Experiment 1 results, haloperidol 1 mg/kg was selected for the evaluations in Experiments 2 and 3. Also, two timespoints were selected that would serve for the baseline (without stimulation) and test (application of aversive stimuli) evaluations in Experiments 2 and 3.

### Experiment 2: Effects of Unconditioned Aversive Stimuli on Haloperidol-Induced Catalepsy

For Experiment 2, a total of 38 animals were sampled. To analyze the influence of immediate exposure to an unconditioned aversive stimulus on haloperidol-induced catalepsy, distinct groups of animals were exposed to a white light (100 lux) or a footshock (0.6 mA) while the step-down latency was measured. Two catalepsy tests were performed, 75 and 90 min after the administration of haloperidol 1 mg/kg. At the beginning of the experiment, rats received an intraperitoneal injection of haloperidol and were immediately placed into the experimental chamber. After 75 min, rats underwent the first catalepsy test (baseline), performed without exposure to any aversive stimuli. The next trial was performed after 15 min (90 min after haloperidol administration): this catalepsy test was performed concomitantly with the presentation of one of the unconditioned aversive stimuli, light or footshock, according to the respective group. Thus, after 20 s of permanence on the horizontal bar, the stimulus was applied. The footshock was delivered for 1 s (*n* = 10), while the light stimulus was presented for 1 s (*n* = 9) or 20 s (*n* = 10). For the non-stimulated group (*n* = 9), the second evaluation of catalepsy was performed without exposure to any aversive stimulus.

### Experiment 3: Effects of Aversive Light Conditioned Stimuli on Haloperidol-Induced Catalepsy

For Experiment 3, a total of 28 animals were sampled. A light-CS was used to evaluate the effects of an aversive conditioned stimulus on haloperidol-induced catalepsy. The study consisted of two sessions: training and testing. Across sessions, the experimental chamber was modified to create two different contexts. During the training session, the elevated bar was removed from the experimental chamber and an acetic acid (2%) odor was added to the chamber. No haloperidol was administered. At the beginning of the session, rats were placed into the experimental chamber and allowed to habituate for 5 min. After habituation, animals received the light stimuli (100 lux) paired (Light-paired group; *n* = 13) or interspersed (Light-unpaired group; *n* = 15) with the unconditioned stimuli footshocks (0.6 mA). The paired group received 10 presentations of light, lasting 20 s each, that ended with a 1-s footshock, with a 30–90 s variable interval. The unpaired group received 10 presentations of light, lasting 20 s each, interspersed with 10 presentations of 1-s footshocks, with a 15–45 s variable interval (De Oliveira et al., 2011; Reimer et al., 2012; De Vita et al., 2021).

Twenty-four hours later, animals were submitted to the test session, in which the catalepsy was evaluated concomitantly with the presentation of the light-CS–no footshocks were administered during this session. For this session, the experimental cage was enclosed in a different sound-attenuating chamber; the floor was covered with a polypropylene plate, and alcohol (20%, used during cleaning) was used as a mild scent. The protocol for the evaluation of catalepsy was the same used in the previous experiments. Initially, animals received an intraperitoneal administration of haloperidol 1 mg/kg and were placed in the experimental chamber. Next, 75 min later, rats underwent the first catalepsy test, which served as the baseline. After a 15 min interval–90 min after haloperidol administration–the second catalepsy test was performed concomitantly with the presentation of light-CS. Thus, after 20 s of permanence on the horizontal bar, the light stimulus was presented for 20 s and the step-down latency was measured. All animals were submitted to a single session, according to their respective group. Light intensity and duration were based on the results of Experiment 2, which showed that light by itself, was not able to affect the catalepsy.

### Data Analysis

Statistical analysis was performed using R (version 4.0.5, R Core Team, 2021) with the package lme4 (version 1.1-26; Bates et al., 2015). For Experiment 1, a generalized linear mixed model (GLMM) was used to account for the repeated-measures design and non-Gaussian distribution of the data; a GLMM with log link function and Gamma distribution was used. Rats’ identities were used as random factors and Treatment (Control, Halo1, and Halo2) and Time as fixed factors. For Experiments 2 and 3 general linear models (GLMs) were used to evaluate step-down response; Group was used as a fixed factor and the contribution of baseline responding (step-down latency at 75-min post-haloperidol treatment) was included as a covariate. For Experiment 3, a GLM was used to evaluate freezing response; Group (Light-unpaired and Light-Paired) was used as a fixed factor.

## Results

### Experiment 1: Evaluation of Haloperidol-Induced Catalepsy Over Time in Rats

**FIGURE 1:**
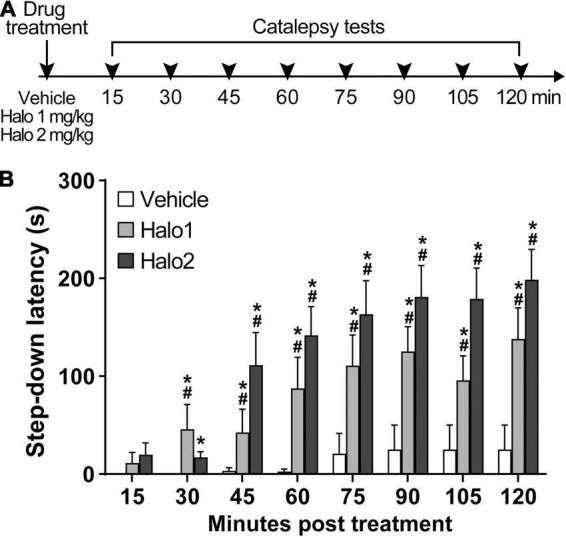
Haloperidol 1 and 2 mg/kg induce long lasting catalepsy when injected i.p. **(A)** Timeline of the experimental procedure. **(B)** Step-down latency in groups of rats receiving haloperidol in doses of 1 (Halo1) or 2 mg/kg (Halo2), or vehicle, evaluated after 15, 30, 45, 60, 75, 90, 105, and 120 min after drug administration. Mean + S.E. *Different from the vehicle-treated group in the same time-point. ^#^Different from the same treatment group at 15 min. *n* = 12 per group.

Both haloperidol 1 and 2 mg/kg induced catalepsy throughout the test (Figure 1). An analysis of variance based on mixed gamma regression indicated statistically significant effect for Treatment (χ^2^ = 80.47, *p* < 0.05), Time (χ^2^ = 156.28, *p* < 0.05), and Treatment × Time interaction (χ^2^ = 82.22, *p* < 0.05). Pairwise comparisons using *Z*-tests, corrected with Holm’s sequential Bonferroni procedure, indicated that haloperidol 1 mg increased step-down latency 30 min (*Z* = −4.12, *p* < 0.05), 45 min (*Z* = −4.21, *p* < 0.05), 60 min (*Z* = −6.11, *p* < 0.05), 75 min (*Z* = −7.45, *p* < 0.05), 90 min (*Z* = −8.34, *p* < 0.05), 105 min (*Z* = −7.51, *p* < 0.05), and 120 min (*Z* = −8.74, *p* < 0.05) after administration, when compared with the same treatment at 15 min. Haloperidol 2 mg/kg increased step-down latency 45 min (*Z* = −5.11, *p* < 0.05), 60 min (*Z* = −6.00, *p* < 0.05), 75 min (*Z* = −6.45, *p* < 0.05), 90 min (*Z* = −6.80, *p* < 0.05), 105 min (*Z* = −6.85, *p* < 0.05), and 120 min (*Z* = −7.12, *p* < 0.05) after administration, when compared with the same treatment at 15 min. Pairwise comparisons also indicated statistical difference between haloperidol 1 mg/kg and vehicle-treated groups at 30 min (*Z* = 4.38, *p* < 0.05), 45 min (*Z* = 4.02, *p* < 0.05), 60 min (*Z* = 5.62, *p* < 0.05), 75 min (*Z* = 6.85, *p* < 0.05), 90 min (*Z* = 7.36, *p* < 0.05), 105 min (*Z* = 6.78, *p* < 0.05), and 120 min (*Z* = 7.52, *p* < 0.05) timespoints; statistical differences were also found between haloperidol 2 mg/kg and vehicle-treated groups at 30 min (*Z* = 3.62, *p* < 0.05), 45 min (*Z* = 6.00, *p* < 0.05), 60 min (*Z* = 7.08, *p* < 0.05), 75 min (*Z* = 7.74, *p* < 0.05), 90 min (*Z* = 7.95, *p* < 0.05), 105 min (*Z* = 7.97, *p* < 0.05), and 120 min (*Z* = 8.19, *p* < 0.05) timespoints. No differences were found between haloperidol 1 and haloperidol 2 groups (*p* > 0.05).

### Experiment 2: Effects of Unconditioned Aversive Stimuli on Haloperidol-Induced Catalepsy

**FIGURE 2:**
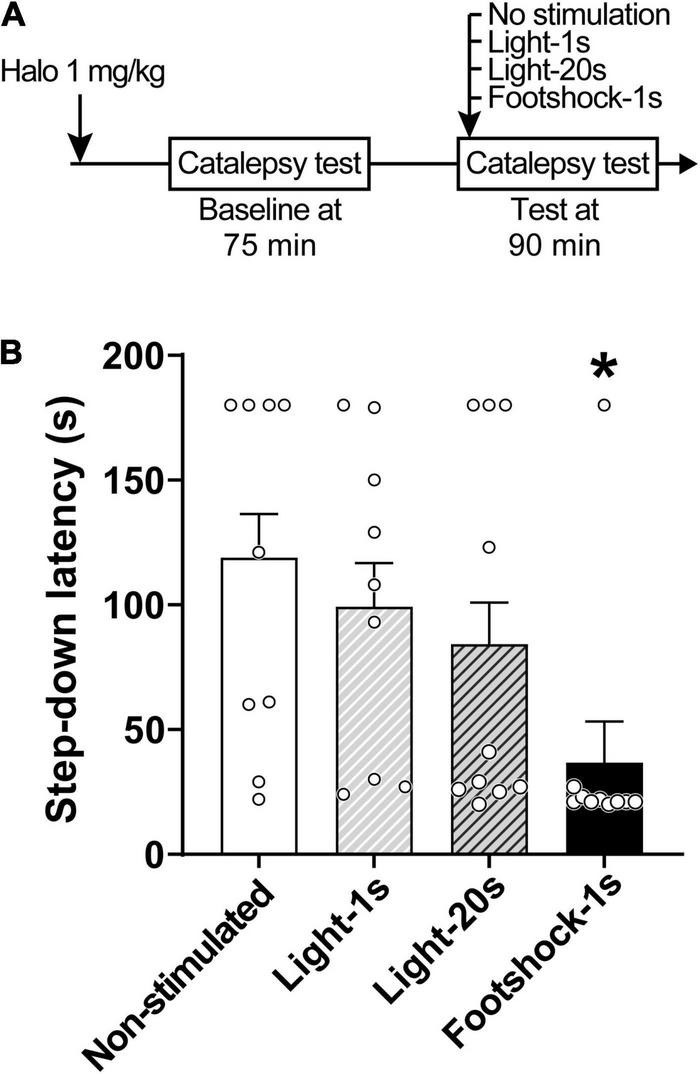
Footshocks reduce step-down latency in the catalepsy test. **(A)** Timeline of the experimental procedure. **(B)** Step-down latency in groups of rats that received no stimulation (Non-stimulated), were stimulated with a 1-s light stimulus (Light-1 s), a 20-s light stimulus (Light-20 s), or a 1-s footshock (Footshock-1 s), 90 min after treatment with haloperidol 1 mg/kg. Marginal estimated means + S.E; circles represent individual animals. *Different from the Non-stimulated group. *n* = 9 for Non-stimulated and Light-1 s groups; *n* = 10 for Footshock-1 s and Light-20 s groups.

Discrete footshocks disrupted the cataleptic state (Figure 2). A linear model analysis of variance indicated a statistically significant effect for Group [*F*_(3,30)_ = 4.29, *p* < 0.05]. Pairwise comparisons using *Z*-tests, corrected with Holm’s sequential Bonferroni procedure, indicated that only Footshocks significantly reduced step-down latency (*Z* = 3.41, *p* < 0.05) when compared to the Non-stimulated group.

### Experiment 3: Effects of Aversive Light Conditioned Stimuli on Haloperidol-Induced Catalepsy

**FIGURE 3:**
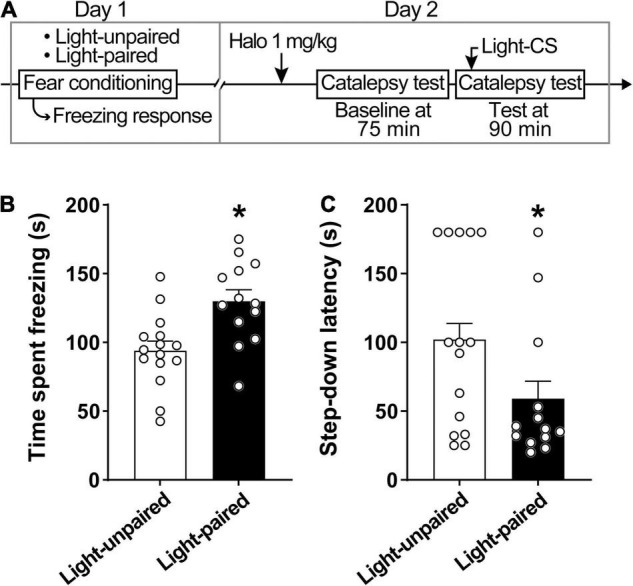
Explicit paired light-CS disrupts haloperidol-induced catalepsy. **(A)** Timeline of the experimental procedure. **(B)** Freezing response during fear acquisition session in rats receiving light-CS and footshock-US paired explicitly (Light-paired), or randomly presented (Light-unpaired). **(C)** Effects of explicit paired Light-CS or unpaired on haloperidol-induced catalepsy, verified 90 after drug administration (1 mg/kg haloperidol). Marginal estimated means + S.E; circles represent individual animals. *Different from the Light-unpaired group. *n* = 15 for Light-unpaired group; *n* = 13 for Light-paired group.

Explicit paired light-CS disrupts haloperidol-induced catalepsy (Figure 3). Rats displayed increased conditioned freezing response when exposed to paired light-CS (Figure 3B). A linear model analysis of variance indicated a statistically significant effect for Group [*F*_(1,26)_ = 11.20, *p* < 0.05], with rats that received paired light-CS exhibiting increased freezing response during fear conditioning when compared with the Light-unpaired group. For catalepsy (Figure 3C), a linear model analysis of variance indicated a statistically significant effect for Group [*F*_(1,24)_ = 6.10, *p* < 0.05], with the Light-paired group exhibiting reduced step-down latency, when compared to the Light-unpaired group.

## Discussion

In the present study, we investigated the immediate effects of unconditioned (light and footshocks) and conditioned (light-CS) aversive stimulation on catalepsy. We confirmed that both haloperidol 1 and 2 mg/kg induce a long-lasting cataleptic state. We also verified that haloperidol-induced catalepsy seems to be differently influenced depending on the modality of the aversive stimulation. Exposure to footshocks and paired light-CS, but not to light-US, significantly reduced step-down latency during the catalepsy test. These results demonstrate face validity for the model proposed here for the study of the paradoxical kinesia phenomenon of PD, wherein patients are able to overcome bradykinesia (Glickstein and Stein, 1991; Jankovic, 2008).

In agreement with a substantial body of evidence, catalepsy was the most prevalent response produced by both haloperidol 1 and 2 mg/kg (Lorenc-Koci et al., 1996; Wadenberg et al., 2001; Greco et al., 2010; Colombo et al., 2013; Barroca et al., 2019). Haloperidol 1 and 2 mg/kg caused significant catalepsy starting 30 min after administration and lasting at least up to 120 min, the endpoint of evaluation in our study. These results confirm previous reports of the cataleptic effect of haloperidol lasting for at least 120 min after administration (Greco et al., 2010), being able to last up to 240–300 min after administration (Verhagen-Kamerbeek et al., 1993; Moo-Puc et al., 2003; Ardashov et al., 2011; Ionov and Severtsev, 2012; Pavlova et al., 2014). The vehicle-treated group did not exhibit catalepsy over time, which is in agreement with evidence in the literature (Colombo et al., 2013; Barroca et al., 2019; Sita et al., 2020), and attests that catalepsy is not predominant in the behavioral repertoire of rats even after multiple exposures to the test. Since catalepsy was successfully achieved with haloperidol 1 mg/kg for the entire period of testing, and this dose is widely used in the literature (Waku et al., 2021), we considered haloperidol 1 mg/kg-induced catalepsy sufficient to assess the effects of aversive stimuli for the following experiments.

Exposure to footshocks was able to reduce the cataleptic state. This result confirms our initial hypothesis that the brief presentation of an aversive stimulus during the catalepsy test would disturb immobility, modeling the paradoxical kinesia phenomenon. Exposure of rats to a water tank or bath of ice (Keefe et al., 1989), microinjection of NMDA antagonists into the inferior colliculus (Melo et al., 2010) and deep brain stimulation of the inferior colliculus (Melo-Thomas and Thomas, 2015), have also been reported to reduce catalepsy or induce movements such as swimming and escape. On the other hand, and contrary to what was expected, exposure to light (for 1 or 20 s) didn’t affect catalepsy. But rather than an isolated event, these results are consistent with a pilot experiment in which the same, and even a lower haloperidol dose (0.5 and 1.0 mg/kg), and a slightly different time window for testing was used (see Supplementary Figure 1). One possibility is that the neural substrate activated in the presence of footshock differs from that associated with an aversive light, with only the former disrupting catalepsy. However, we cannot rule out that visual stimuli are capable of inducing paradoxical kinesia, particularly as several clinical reports indicate that objects/people in motion and visual cues can elicit sudden movement in Parkinsonian patients (Glickstein and Stein, 1991; Jankovic, 2008). Using a switch-off paradigm, Reis et al. (2004) and Saito and Brandão (2016) showed that rats actively avoid a 120 lux light, which is slightly higher than the light intensity used in this work (100 lux). Similarly, Barker et al. (2010) reported that rats prefer food alone over food plus light in an operant conditioning paradigm when high-intensity stimuli are used (8,180 lux). Considering that the light aversiveness is dependent on stimulus duration, intensity, and the experimental context (Barker et al., 2010; Saito and Brandão, 2016), we cannot rule out the possibility that the light used here did not provide sufficient aversion to disturb catalepsy.

We should also consider that the light stimulus is often used as a conditioned stimulus (De Oliveira et al., 2006, 2017; Cassaday and Thur, 2015; Pezze et al., 2016). In the context of PD research, however, little is known regarding how conditioned stimuli affect paradoxical kinesia. Accordingly, we evaluated aversive light-CS as a potential disruptor for the haloperidol-induced catalepsy in rats. We expected and later confirmed that brief reexposure to light-CS during the catalepsy test would disrupt immobility, mimicking the paradoxical kinesia in PD. McDonald et al. (2015) observed a similar effect in PD patients, who, when motivated to avoid mild electric shock, showed shorter reaction times to start a motor response. Schlesinger et al. (2007) showed, however, that powerful, frightening auditory cues warning of rockets strike in a war zone were not enough to induce paradoxical kinesia (Schlesinger et al., 2007).

In general, our results suggest that aversive unconditioned–footshock–and conditioned stimulus–light-CS–when present during the catalepsy test, can disrupt the cataleptic state. Such results suggest face validity for the model proposed here for the study of paradoxical kinesia using immediate exposure to these aversive stimuli. Indeed, fear and anxiety motivate behaviors that aim to reduce potential dangers, such as arousal and escape behaviors (McNaughton and Corr, 2004; Brandão et al., 2005; Mobbs et al., 2009). Thus, immediate exposure to aversive stimulation may trigger sudden motor responses, resembling patients with PD when exposed to situations that generate intense emotions, such as fear.

In PD, motor impairments are due to difficulties in selecting and executing motor actions, associated with dopamine loss in basal ganglia causing excessive inhibitory output to the thalamo-cortical system and brainstem, leading to reduced activation of motor centers (Takakusaki et al., 2003, 2004; Naugle et al., 2010; Dickson, 2012). Considering that motor and affective limbic networks seem to be integrated *via* a striato-nigro-striatal network (Bjorklund and Dunnett, 2007; Haber and Calzavara, 2009), it is not surprising that the motor impairments in PD can be influenced by the patient’s emotional state. Paradoxical kinesia is generally thought to be mediated either by activation of basal ganglia dopaminergic reserves, by compensatory activation of alternative pathways, or *via* noradrenergic augmentation (Glickstein and Stein, 1991; Schlesinger et al., 2007; McDonald et al., 2015). Activation of basal ganglia reserves is supported by studies showing increased dopamine release in the striatum in PD patients (De la Fuente-Fernández et al., 2001a,b; Martinez et al., 2013). In fact, PD patients have the greatest functional deficit in the posterior putamen, while keeping the potential to use the rostro-medial striatum and the limbic territories of the basal ganglia (Redgrave et al., 2010; Nonnekes et al., 2019). Dopamine may contribute to the phenomenon of paradoxical kinesia, considering that dopaminergic mechanisms take part in regulating adaptive responses to aversive situations (De Oliveira et al., 2014; Brandão et al., 2015). Under aversive conditions, increases in residual dopaminergic neurons firing rate in limbic parts of the basal ganglia could occur, allowing unconstrained movement (Duysens and Nonnekes, 2021). Consistent with this hypothesis, in response to aversive stimulation, several authors have reported increased activation of substantia nigra and ventral tegmental area neurons and release of dopamine in the striatum (Chiodo et al., 1979, 1980; Keller et al., 1983; Abercrombie et al., 1989; Martinez et al., 2008).

However, other evidence from animal models seems to favor a non-dopaminergic mechanism for the phenomenon of paradoxical kinesia. Rats cataleptic through administration of 6-hydroxydopamine and subsequently treated with haloperidol, exhibit swimming behavior when placed in a deep water tank and escape when placed in a shallow water ice bath (Keefe et al., 1989), pointing to the involvement of circuits other than dopaminergic in this phenomenon. Activation of the noradrenergic system could act to facilitate information flow through the striatum when dopamine-depleted subjects are exposed to aversive stimulation enabling them to overcome their motor deficit (Brown and Marsden, 1998; Cunnington et al., 1999). Paradoxical kinesia may also occur through alternative neural pathways, such as cerebellar circuitries (Glickstein and Stein, 1991; Hanakawa et al., 1999; Goerendt et al., 2004; Loayza et al., 2022), hippocampal/amygdala-accumbens-subpallidal pathway (Mogenson and Nielsen, 1984; Morrison et al., 2017), and inferior colliculus (Melo et al., 2010; Melo-Thomas and Thomas, 2015).

In PD research, animal models continue to be essential for unraveling pathophysiologic mechanisms as well as treatment optimization. Animal models for PD have accelerated progress toward better dopaminergic drug treatments and minimizing drug-related side effects (Duty and Jenner, 2011). In parallel, animal models contributed and continue allowing opportunities to develop and improve therapies such as cannabidiol-based treatments (Crippa et al., 2018) and deep brain stimulation interventions (Knorr et al., 2022). Here, haloperidol-induced catalepsy was reduced with immediate exposure to the unconditioned aversive stimulus footshock or a light previously associated with the presentation of footshocks, but not with exposure to an unconditioned 100 lux light lasting 1 or 20 s. Catalepsy appears to be differently influenced depending on the modality of the aversive stimulation used. The selective recruitment of threat response systems may bypass the dysfunctional motor circuit, leading to the activation of alternative routes to drive movement. Future investigation of the neural substrates of the observed emotional modulation of catalepsy will clarify if the footshock and light-CS-induced disruption of catalepsy are similarly mediated. The results also point to the face validity of a promising animal model for the study of paradoxical kinesia that uses exposure to immediate moderate aversive stimuli to disrupt haloperidol-induced catalepsy in rats. Continued work in this area could contribute to the understanding of the neural bases associated with the paradoxical kinesia phenomenon and the interactions between emotion and movement, favoring the improvement of therapies for PD and related disorders.

## Data Availability Statement

The raw data supporting the conclusions of this article will be made available by the authors, without undue reservation.

## Ethics Statement

The animal study was reviewed and approved by Committee for Animal Care and Use of the Federal University of São Carlos (protocol no. 5413130319).

## Author Contributions

AO designed the study and supervised the project. IW performed the research. AR compiled the figures. AO, AR, and IW wrote the manuscript. All authors approved the final version of the manuscript.

## Conflict of Interest

The authors declare that the research was conducted in the absence of any commercial or financial relationships that could be construed as a potential conflict of interest.

## Publisher’s Note

All claims expressed in this article are solely those of the authors and do not necessarily represent those of their affiliated organizations, or those of the publisher, the editors and the reviewers. Any product that may be evaluated in this article, or claim that may be made by its manufacturer, is not guaranteed or endorsed by the publisher.
