# Supplementary material for: Effects of Immediate Aversive Stimulation on Haloperidol-Induced Catalepsy in Rats
Source: Front Behav Neurosci. 2022 Apr 11;16:867180. doi: 10.3389/fnbeh.2022.867180 (PMC9036068; doi:10.3389/fnbeh.2022.867180)
Supplement: Supplementary file 1 [file Data_Sheet_1.PDF]

Supplementary Material

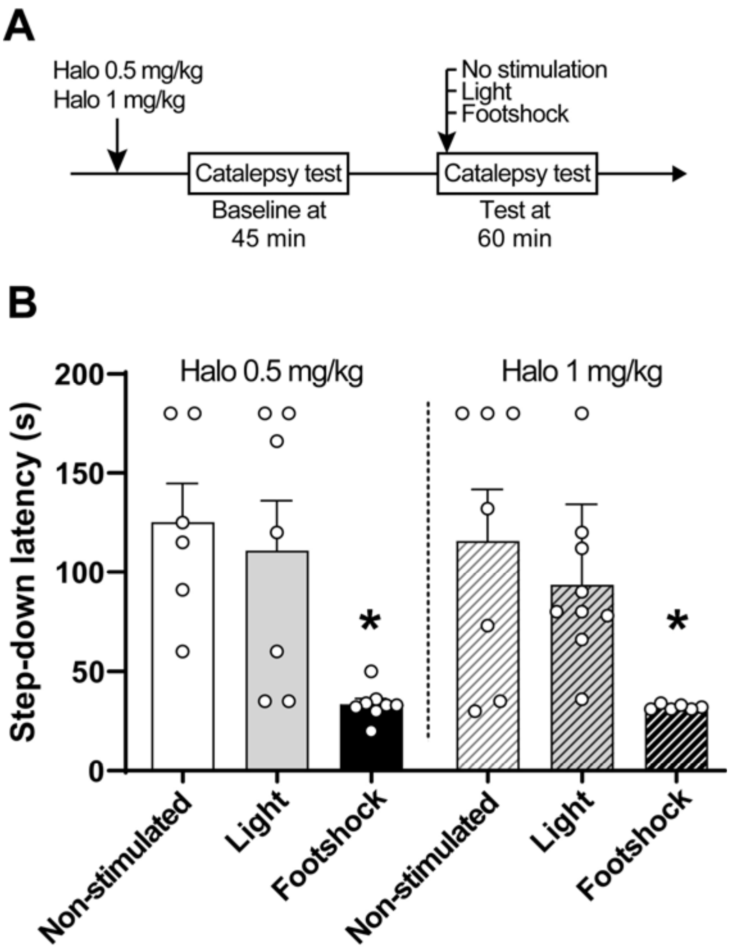

Supplementary Figure 1. Footshocks reduce step-down latency in the catalepsy test. A. Timeline of the experimental procedure. **B.** Step-down latency in groups of rats that received no stimulation (Non-stimulated), were stimulated with a 1-s light stimulus (Light), or a 1-s footshock (Footshock), 60 min after treatment with haloperidol 0.5 or 1 mg/kg (Teuto, Brazil). Pairwise comparisons using Z-tests, corrected with Holm's sequential Bonferroni procedure, indicated that Footshocks significantly reduced step-down latency (halo 0.5: $Z = -4.31$, $p < 0.05$; halo 1: $Z = -3.51$, $p < 0.05$), when compared to the Non-stimulated group. Marginal estimated means+S.E; circles represent individual animals. * Different from the Non-stimulated group. n = 6 for Halo 1-Footshock and Halo 0.5-Non-stimulated; n = 7 for Halo 1-Non-stimulated and Halo 0.5-Light; n = 8 for Halo 0.5-Footshock; n = 9 for Halo 1-Light.
